# Collaborative Cross Mice Yield Genetic Modifiers for Pseudomonas aeruginosa Infection in Human Lung Disease

**DOI:** 10.1128/mBio.00097-20

**Published:** 2020-03-03

**Authors:** Nicola Ivan Lorè, Barbara Sipione, Gengming He, Lisa J. Strug, Hanifa J. Atamni, Alexandra Dorman, Richard Mott, Fuad A. Iraqi, Alessandra Bragonzi

**Affiliations:** a Infections and Cystic Fibrosis Unit, Division of Immunology, Transplantation and Infectious Diseases, IRCCS San Raffaele Scientific Institute, Milan, Italy; b Vita-Salute San Raffaele University, Milan, Italy; c Program in Genetics and Genome Biology, The Hospital for Sick Children Research Institute, Toronto, Ontario, Canada; d Division of Biostatistics, Dalla Lana School of Public Health, University of Toronto, Toronto, Ontario, Canada; e Department of Clinical Microbiology and Immunology, Sackler Faculty of Medicine, Tel Aviv University, Tel Aviv, Israel; f Genetics Institute, University College London, London, United Kingdom; Emory University School of Medicine

**Keywords:** *Pseudomonas aeruginosa*, gene modifiers, respiratory infection, mouse model

## Abstract

Human genetics influence a range of pathological and clinical phenotypes in respiratory infections; however, the contributions of disease modifiers remain underappreciated. We exploited the Collaborative Cross (CC) mouse genetic-reference population to map genetic modifiers that affect the severity of Pseudomonas aeruginosa lung infection. Screening for P. aeruginosa respiratory infection in a cohort of 39 CC lines exhibits distinct disease phenotypes ranging from complete resistance to lethal disease. Based on major changes in the survival times, a quantitative-trait locus (QTL) was mapped on murine chromosome 3 to the genomic interval of Mb 110.4 to 120.5. Within this locus, composed of 31 protein-coding genes, two candidate genes, namely, dihydropyrimidine dehydrogenase (*Dpyd*) and sphingosine-1-phosphate receptor 1 (*S1pr1*), were identified according to the level of genome-wide significance and disease gene prioritization. Functional validation of the *S1pr1* gene by pharmacological targeting in C57BL/6NCrl mice confirmed its relevance in P. aeruginosa pathophysiology. However, in a cohort of Canadian patients with cystic fibrosis (CF) disease, regional genetic-association analysis of the syntenic human locus on chromosome 1 (Mb 97.0 to 105.0) identified two single-nucleotide polymorphisms (rs10875080 and rs11582736) annotated to the *Dpyd* gene that were significantly associated with age at first P. aeruginosa infection. Thus, there is evidence that both genes might be implicated in this disease. Our results demonstrate that the discovery of murine modifier loci may generate information that is relevant to human disease progression.

## Abstract (precis)

Respiratory infection caused by P. aeruginosa is one of the most critical health burdens worldwide. People affected by P. aeruginosa infection include patients with a weakened immune system, such as those with cystic fibrosis (CF) genetic disease or non-CF bronchiectasis. Disease outcomes range from fatal pneumonia to chronic life-threatening infection and inflammation leading to the progressive deterioration of pulmonary function. The development of these respiratory infections is mediated by multiple causes. However, the genetic factors underlying infection susceptibility are poorly known and difficult to predict. Our study employed novel approaches and improved mouse disease models to identify genetic modifiers that affect the severity of P. aeruginosa lung infection. We identified candidate genes to enhance our understanding of P. aeruginosa infection in humans and provide a proof of concept that could be exploited for other human pathologies mediated by bacterial infection.

## INTRODUCTION

Identifying host genetic factors that contribute to susceptibility to respiratory infection remains a central challenge for understanding disease processes and can provide new insights toward precision medicine. In humans, the identification of disease modifiers is a lengthy and expensive endeavor with an unpredictable success rate. Therefore, novel approaches and improved disease models are required to move the field forward.

Among respiratory infections, those caused by the opportunistic pathogen Pseudomonas aeruginosa retain critical roles in clinical settings (1, 2). P. aeruginosa infections are associated with bacterial pneumonia in patients with a range of pathological and clinical phenotypes owing to different etiologies and underlying conditions, such as cystic fibrosis (CF) and chronic obstructive pulmonary disease (COPD). According to the latest World Health Organization (WHO) estimates, 64 million people worldwide have COPD, and 70,000 have CF (http://www.who.int/).

The outcomes of P. aeruginosa infections in patients with chronic respiratory illnesses are difficult to predict and extremely variable, with the severity of the pulmonary conditions ranging from mild to life-threatening. Recent studies suggest that these outcomes are mediated not only by the presence of the microbes *per se* but also by host responses. In particular, host genetic factors, such as the involvement of multiple genetic loci and the presence of polymorphic variants, influence pathological traits, including airway pathophysiology and P. aeruginosa infection (3, 4). The most striking example is CF, in which obstructive lung disease is not exclusively correlated with mutations in the disease-causing gene *Cystic Fibrosis transmembrane conductance regulator* (*CFTR*); individuals sharing the same *CFTR* variants exhibit substantial variation in the severity of lung disease, >50% of which is explained by non-*CFTR* genetic variations (5). However, identifying genetic disease modifiers by genome-wide association studies (GWASs) in humans requires a very large cohort of subjects with detailed phenotypes in order to achieve statistical significance (6).

Since human studies are time-consuming and costly, we have investigated whether disease modifiers can be identified in animal models. Collaborative Cross (CC) mice provide a useful tool to model human disease heterogeneity in terms of genetic diversity. The chromosomes of each inbred recombinant CC line are a genetic mosaic from eight founder haplotypes (7). Three of the founders (A/J, C57BL/6J, and 129S1/SvImJ) are common laboratory strains; three strains (CAST/EiJ, PWK/PhJ, and WSB/EiJ) are wild derived, ensuring maximum genetic diversity; and two strains (NOD/LtJ and NZO/HiLtJ) carry genetic variants predisposing to medically important conditions. Consequently, CC mice have greater recombination and genetic variation (90%) than other commercially available reference panels (30%) (8). High mapping resolution and sufficient sample sizes to drive phenotypic diversity in almost any trait of interest are hallmarks of CC lines. Simulations and previous mapping studies indicate that the use of the CC population can enable mapping of quantitative-trait loci (QTLs) of about 1 Mb (8, 9). CC mice show a spectrum of pathogenic phenotypes absent in classical inbred strains, making them particularly useful for forward-genetics approaches. Pathological manifestations across CC lines after infection are similar in variety and proportion to the spectra of clinical diseases caused by Ebola virus (10), Mycobacterium tuberculosis (11), West Nile virus (12), severe acute respiratory syndrome (SARS) coronavirus (13), and influenza A virus (14). The power and resolution of mapping with CC mice have been demonstrated for susceptibility to Aspergillus fumigatus (9), Klebsiella pneumoniae (15), influenza A (14), and SARS coronavirus (13). However, at the time of writing, none of these previous findings in CC mice have yet been validated functionally and translated to humans.

We previously demonstrated that the genetically diverse CC mouse population displays a wide range of heritable responses to respiratory infection with P. aeruginosa (16). Thus, genetic disease modifiers likely influence morbidity and mortality from P. aeruginosa pneumonia, but they remain to be identified and translated to humans. Here, we used CC mice and a disease model to map genetic modifiers of P. aeruginosa respiratory infection. Functional validation of a selected disease gene confirmed its relevance in P. aeruginosa pathophysiology. By using the conserved synteny between mouse and human, and data derived from a cohort of patients infected by P. aeruginosa, we identified human candidate genes within the corresponding syntenic human locus. Our data demonstrate that this approach generates new insights and translational knowledge about genetic modifiers of human disease.

## RESULTS

### CC lines display a wide range of disease-related phenotypes during P. aeruginosa respiratory infection.

**FIG 1:**
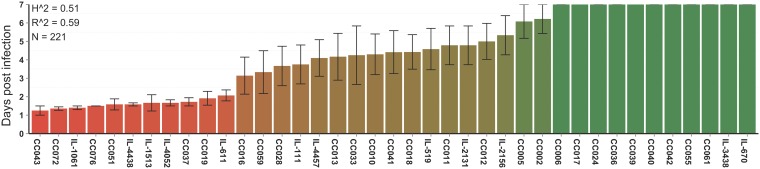
Phenotypic profile of mean survival time in CC lines after P. aeruginosa airway infection. Mice from 39 CC lines were infected with 1 × 10^6^ CFU per animal of the planktonic P. aeruginosa AA2 clinical strain by intratracheal injection and monitored for mean survival time over 7 days (*n *= 221, including 100 female and 121 male mice). Broad-sense heritability (Ĥ2) and predictive accuracy (R̂2) are shown. Mice are represented with a scale color from red to green associated with the variation of phenotypic traits.

Systematic screening for susceptibility to P. aeruginosa infection was carried out in 39 CC lines, which included a total of 221 mice (100 female and 121 male) aged between 7 and 14 weeks. Human bronchopulmonary infection was mimicked in mice by exposing the murine airways to the P. aeruginosa AA2 clinical strain by intratracheal (i.t.) injection, as previously described (17). CC lines were monitored for mortality (survival time). Segregation analysis of the phenotypic-trait distribution was carried out to score different CC mouse strains according to the mean survival time over 7 days (Fig. 1; see also Data Set S1 in the supplemental material). CC lines exhibited highly variable susceptibility to P. aeruginosa infection ranging from complete resistance to lethal disease.

10.1128/mBio.00097-20.10 DATA SET S1 Raw data of CC mouse strains, including age, gender, initial body weight, and survival time after P. aeruginosa infection. Download Data Set S1, CSV file, 0.01 MB. Copyright © 2020 Loré et al. 2020 Loré et al. This content is distributed under the terms of the Creative Commons Attribution 4.0 International license.

To test whether the trait variations within the population of infected mice were the result of genetic factors, we estimated broad-sense heritability (*H*^2^) as previously described (16). We obtained values of 0.51 for the mean survival time (Fig. 1). These data confirm that the broad range of responses to P. aeruginosa infection in CC lines is affected by their genetic variability.

### Mapping QTLs for the severity of P. aeruginosa respiratory infection.

**FIG 2:**
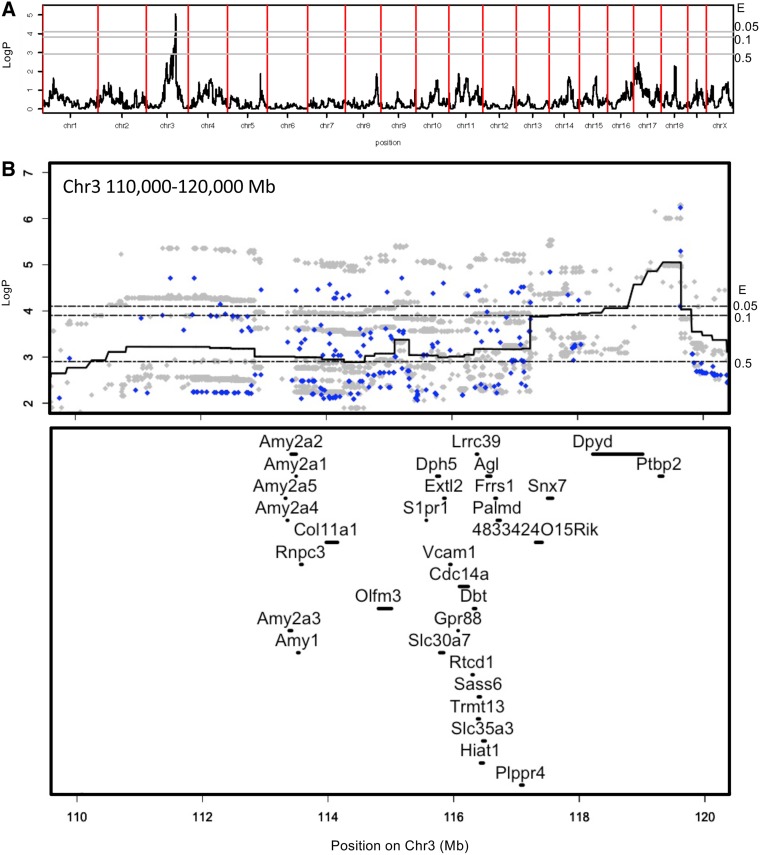
Identification of QTLs associated with mean survival time on day 7 after P. aeruginosa infection. (A) Genome location (*x* axis) and log*P* of the test of association between locus and survival time (*y* axis). Genome-wide thresholds of association at expectation levels of an *E* value of <0.5, an *E* value of <0.1, and an *E* value of <0.05 are indicated by the horizontal gray lines at log*P* values of 2.9, 3.9, and 4.1, respectively (i.e., the threshold *P* means that in a fraction, *P*, of permutations, the genome-wide maximum log*P* exceeded the threshold). (B) Merge analysis of the chromosome 3 QTL between Mb 105 and Mb 125. The *x* axis is the genome location, and the *y* axis is the log*P* of the test of association between locus and survival time. The continuous black line is the genome scan. The blue (biallelic) and gray (multiallelic) dots are the results of merge analyses of sequence variants segregating in the eight founders of the CC. Moreover, the genomic positions of genes located in the murine QTL region are shown.

To identify genetic loci associated with P. aeruginosa infection, we performed genome-wide association analysis with publicly available, high-density single-nucleotide polymorphism (SNP) genotype data for CC mice using HAPPY software based on the R language (9, 15, 18–20). Because of the high levels of homozygosity in each line, the use of genotypes from single representatives of each line is sufficient for QTL mapping purposes (9, 15, 18, 21). A logistic regression model was used to evaluate the effects of gender, batch, and initial body weight on trait distribution. We found that initial body weight significantly affected survival traits, while gender and batch effects did not. We used a haplotype-based test of association to detect QTLs, in which each of the eight CC founder haplotypes was permitted to have a different phenotypic effect. We identified a QTL on chromosome 3 (Chr 3) associated with the mean survival time of P. aeruginosa infection at day 3 postinfection up to day 7, with a peak exceeding the 95% genome-wide permutation-based significance threshold (Fig. S1). In 95% of permuted genome scans, the genome-wide maximum of the negative base 10 logarithm of the *P* value of the test of no association (log*P*) was 4.1; in 90%, it was 3.9; and in 50%, it was 2.9. In 50% of permuted genome scans, this QTL at day 7 postinfection was mapped within the interval of chr3 from Mb 110.4 to 120.5 on NCBI37 (Fig. 2A). Next, we used merge analysis (9, 15, 18, 22, 23) to test the association of individual sequence variants within the QTL segregating between the CC founder strains (Fig. 2B). The SNPs with the strongest associations were both biallelic and multiallelic. However, a merge analysis of the imputed sequence variants under the QTL peak identified 65 SNPs with log*P* values of >5.5. Among these, there are just three distinct founder strain distribution patterns. They all involve a contrast of A/J and CAST/EiJ versus the rest, with the PWK allele being ambiguous. This is consistent with the founder strain patterns. Moreover, the founder allelic effects at the QTL peak, shown in the haplotype dosage plot in Fig. S2, indicate that haplotypes associated with the founders A/J and CAST/EiJ confer resistance, those associated with the founders 129S1/SvImJ and WSB/EiJ confer susceptibility, and those associated with the other founders were midway. This suggests that multiple causal variants might segregate within the QTL, giving rise to haplotypic differences in terms of mean survival time to infection.

10.1128/mBio.00097-20.2 FIG S1 Genome scans and QTLs during P. aeruginosa respiratory infection. QTLs associated with survival time (ST) were detected 2 days (A), 3 days (B), 4 days (C), and 7 days (D) after P. aeruginosa infection in CC mice. The *x* axis is the genome location, and the *y* axis is the log*P* of the test of association between locus and survival time. Genome-wide thresholds of association at expectation levels of an *E* value of <0.5, an *E* value of <0.1, and an *E* value of <0.05 are indicated by the horizontal gray lines in each (i.e., the threshold *P* means that in a fraction, *P*, of permutations, the genome-wide maximum log*P* exceeded the threshold). Download FIG S1, TIF file, 0.2 MB. Copyright © 2020 Loré et al. 2020 Loré et al. This content is distributed under the terms of the Creative Commons Attribution 4.0 International license.

10.1128/mBio.00097-20.3 FIG S2 Survival plot of the allelic founder haplotypes at the peak of the QTL (JAX00112378). The survival plot, categorized using the hard-called founder haplotypes at JAX00112378, shows strong differences between haplotypes. There are probably no CC lines carrying the PWK haplotype at this locus. Download FIG S2, TIF file, 0.2 MB. Copyright © 2020 Loré et al. 2020 Loré et al. This content is distributed under the terms of the Creative Commons Attribution 4.0 International license.

A total of 31 protein-coding sequences were located within the 50% interval of the QTL (chr3 Mb 110.4 to 120.5), of which two genes, *Dpyd* and *Ptbp2*, are within the 5% interval (chr3 Mb 118.8 to 119.6) (Fig. 2B).

### Prioritization and functional validation of candidates for disease modifier genes relevant to P. aeruginosa airway infection.

The 31 genes within the QTL were prioritized by scoring each of them using the most significant *P* value for the QTL containing the gene (one point for each of the 50%, 10%, and 5% significance levels) (Fig. 2B) and for protein expression in human airways, in particular in pneumocytes and bronchial cells (information from the Human Protein Atlas Database) (24) (Table S1). We identified 15 genes that scored 3 or higher as candidates for involvement in P. aeruginosa pneumonia. We then used the Beegle tool for disease gene prioritization (25) (http://beegle.esat.kuleuven.be/) with the query “lung infection” (Table 1). Among others, *S1pr1* (sphingosine-1-phosphate receptor 1) was ranked as one of the most promising candidates. *S1pr1* encodes a G-protein-coupled receptor (S1P1) that binds the bioactive signaling molecule sphingosine-1-phosphate (S1P) and is involved in several physiological processes, including inflammation mediated by viral and bacterial infections (26).

**TABLE 1:** P. aeruginosa modifier candidate genes ranked with the Beegle online tool^*a*^

Rank	Gene	Protein
1	*S1pr1*	Sphingosine-1-phosphate receptor 1
2	*Vcam1*	Vascular cell adhesion protein 1
3	*Col11a1*	Collagen alpha-1(XI) chain
4	*Dpyd*	Dihydropyrimidine dehydrogenase (NADP^+^)
5	*Agl*	Amylo-1,6-glucosidase,4-alpha-glucanotransferase
6	*Cdc14a*	Dual-specificity protein phosphatase CDC14A
7	*Snx7*	Sorting nexin 7
8	*Ptbp2*	Polypyrimidine tract-binding protein 2
9	*Lrrc39*	Leucine-rich-repeat-containing protein 39
10	*SLC30A7*	Solute carrier family 30 (zinc transporter), member 7
11	*Palmd*	Palmdelphin
12	*Dbt*	Dihydrolipoamide branched-chain transacylase
13	*Gpr88*	G-protein-coupled receptor 88
14	*Dph5*	Diphthine methyl ester synthase
15	*Frrs1*	Ferric-chelate reductase 1

a The ranking among the final 15 candidate disease modifier genes was generated by the Beegle informatics tool (http://beegle.esat.kuleuven.be/). Beegle software started by mining the literature to quickly extract a set of genes known to be linked with the query “lung infection” and then train a genomic model and rank the set of the 15 candidate genes within the murine QTL.

10.1128/mBio.00097-20.8 TABLE S1 Prioritization of candidate genes for susceptibility to P. aeruginosa respiratory infection. Download Table S1, DOCX file, 0.03 MB. Copyright © 2020 Loré et al. 2020 Loré et al. This content is distributed under the terms of the Creative Commons Attribution 4.0 International license.

**FIG 3:**
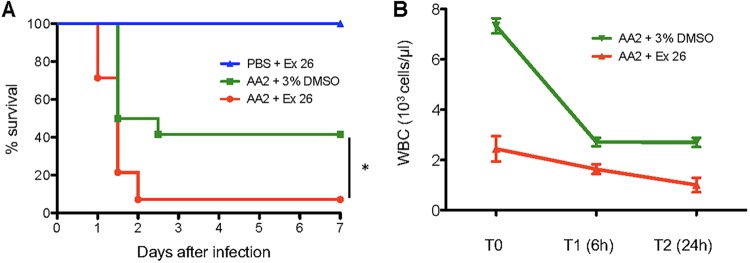
Functional validation of sphingosine-1-phosphate receptor 1 in mice infected with P. aeruginosa. C57BL/6NCrl mice were infected with 5 × 10^6^ CFU of the P. aeruginosa AA2 clinical strain or challenged with PBS as a control. Next, mice were treated with the Ex26 compound at a dose of 3 mg/kg (*n* = 14) or with the 3% DMSO vehicle only (*n* = 12). (A) Survival rates of mice infected with P. aeruginosa and treated with the Ex26 compound or the vehicle or challenged with PBS and treated with the Ex26 compound (*, *P* < 0.05 by a Mantel-Cox test). (B) White blood cell (WBC) count in mice (*n* = 3) at *T*_0_ (before infection), *T*_1_ (6 h), and *T*_2_ (24 h) after infection with P. aeruginosa and Ex26 treatment.

We then tested *S1pr1* as a candidate for involvement in P. aeruginosa infection by functional validation in a mouse model of airway infection. We asked whether perturbation of the *S1pr1* pathway alters the outcome of P. aeruginosa infection, using a selective *S1pr1* antagonist (Ex26) (27) and a functional antagonist that induces *S1pr1* internalization (FTY720) (28). C57BL/6NCrl mice were selected for their intermediate susceptibility to P. aeruginosa infection when challenged with a dose of 5 × 10^6^ CFU of the AA2 strain (17). Under these conditions, between 60 and 75% of untreated C57BL/6NCrl mice died after P. aeruginosa infection, giving a window of opportunity to evaluate either beneficial or detrimental effects of the pharmacological treatment. Treatment of P. aeruginosa-infected mice with Ex26 or FTY720 resulted in significantly higher mortality at 7 days than in mice infected but treated with the corresponding vehicle (93% for Ex26 versus 60% for the control [Ctrl] [*P* < 0.05]; 100% for FTY720 versus 75% for the Ctrl [*P* < 0.05]) (Fig. 3A and Fig. S3). As a control, Ex26 or FTY720 treatment alone in noninfected mice showed no mortality. In addition, treatment with Ex26 and FTY720 reduced the total number of white blood cells (WBC), particularly circulating lymphocytes (Fig. 3B and Fig. S3), demonstrating their effect as immunomodulators. Our results suggest that reduced levels of WBC by the inhibition of *S1pr1* worsen the outcome of P. aeruginosa acute respiratory infection.

10.1128/mBio.00097-20.4 FIG S3 Functional validation of S1Pr1 in mice infected with P. aeruginosa using the FTY720 compound and lymphocyte counts in mice treated with Ex26 and FTY720. C57BL/6N mice were infected with 5 × 10^6^ CFU per animal of the AA2 P. aeruginosa clinical strain or challenged with PBS and treated with FTY720 at a dose of 3 mg/kg (*n* = 12) or with the DMSO vehicle only (*n* = 12). (A) Survival rates of mice infected with P. aeruginosa and treated with FTY720 (red line) or the vehicle (green line) or challenged with PBS and treated with FTY720 (blue line) (*, *P* < 0.05 by a Mantel-Cox test). (B) White blood cell (WBC) count in mice (*n* = 3) at *T*_0_ (before infection), *T*_1_, and *T*_2_ (6 h and 24 h postinfection). (C and D) Total numbers of lymphocytes in mice infected with P. aeruginosa (or challenged with PBS) and treated with FTY720 (C) or Ex26 (D) and the corresponding vehicle. Download FIG S3, TIF file, 0.3 MB. Copyright © 2020 Loré et al. 2020 Loré et al. This content is distributed under the terms of the Creative Commons Attribution 4.0 International license.

### Assessment of disease modifier genes in a cohort of patients with CF phenotyped for age at first P. aeruginosa infection.

**FIG 4:**
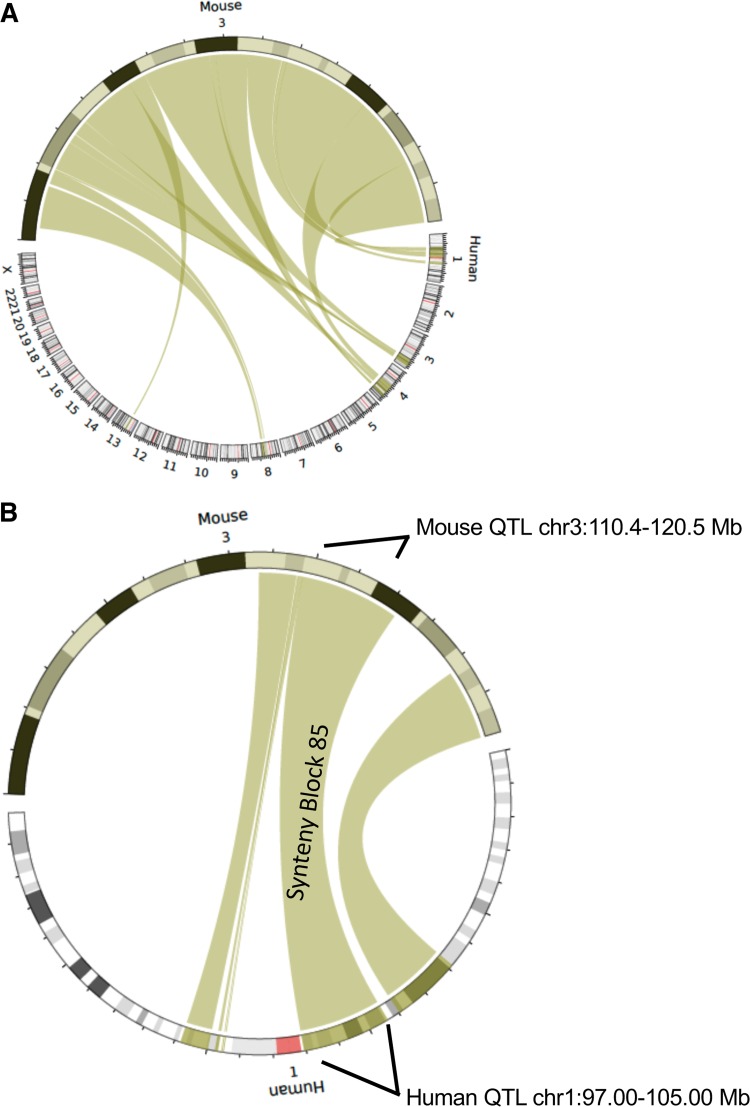
Mouse and human comparative genomics of the identified QTL. (A) Circos plot (generated with the SynCircos tool) showing the alignment of synteny blocks between mouse Chr 3 and human chromosomes. (B) Circos plot showing the alignment of synteny block 85 between mouse Chr 3 (containing the murine QTL that is associated with survival of P. aeruginosa infection) and the homologous human locus on Chr 1.

**FIG 5:**
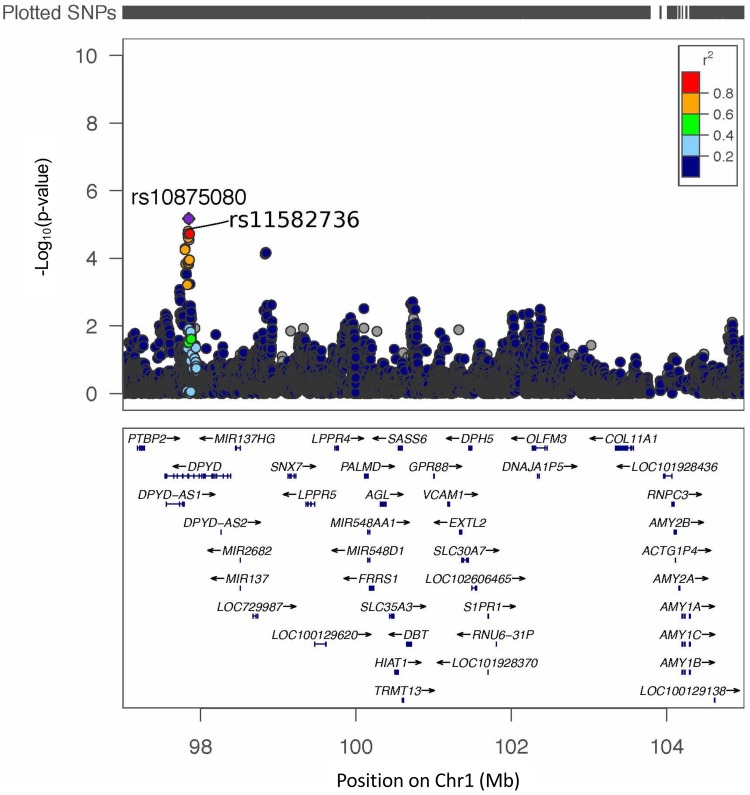
Genetic-association analysis at the human locus syntenic with murine Chr 3 with the age at the first P. aeruginosa infection. Color coding indicates the linkage disequilibrium (*r*^2^) between rs10875080 and the other SNPs analyzed in the region. Association analysis was conducted for the square-root-transformed age at the first P. aeruginosa infection (in 712 individuals with CF) with 17,139 SNPs at the human locus (chromosome 1 Mb 97.0 to 105.0; human genome version 19) syntenic with the murine QTL that is associated survival in the presence of P. aeruginosa infection.

To translate our results to human lung disease, we used Synteny Portal (29) (Fig. 4) to find the human locus syntenic with murine Chr 3 Mb 110.4 to 120.5, which is located at Chr 1 p21.3 to p21.1 (Mb 97.0 to 105.0) on human genome version 19 (hg19) (Fig. S4). We then analyzed a well-characterized patient cohort for P. aeruginosa respiratory infection, the Canadian CF Gene Modifier cohort (30). Clinical microbiological data for P. aeruginosa infection were used to identify candidate genes. Within the syntenic region, we tested for regional, not genome-wide, genetic association using the age at the first P. aeruginosa infection as the phenotype. This is a robust and commonly collected P. aeruginosa phenotype in clinical samples and has been previously shown to be associated with CF modifier genes (30). This primary analysis was followed up by assessing the specificity with other correlated phenotypes, including chronic P. aeruginosa infection (correlation with first P. aeruginosa infection = 0.68; *P* < 2 × 10^−6^) and lung disease severity. Specifically, we tested for an association between the square-root-transformed age at the first P. aeruginosa infection and 17,139 SNPs in Chr 1 Mb 97.0 to 105.0 of hg19 in 712 individuals of European descent with severe *CFTR* genotypes known to be associated with pancreatic insufficiency (Table S2A). The age at the first P. aeruginosa infection ranges from 0.09 years to 20 years. On average, the first P. aeruginosa infection occurs at approximately 6 years of age. Two SNPs reached regional significance: the regression model coefficient for rs10875080 was 0.26, corresponding to a 0.07-year delay of the first P. aeruginosa infection for each additional A allele (*P* = 6.72 × 10^−6^) (minor allele frequency [MAF] = 0.28, corresponding to 51% GG, 42% GA, and 7% AA), and that for rs11582736 was 0.25, corresponding to a 0.07-year delay of the first P. aeruginosa infection for each additional A allele (*P* = 1.56 × 10^−5^) (MAF = 0.24, corresponding to 57% GG, 38% GA, and 5% AA) (Fig. 5 and Table S2B to E). These SNPs are annotated to intron 14 of *DPYD* (Fig. 5). In the smaller sample of individuals for whom age at chronic P. aeruginosa infection was available (*n *= 256), no SNP reached regional significance, although rs10875080 displayed evidence of association at the 5% level (*P* = 0.02; coefficient estimate = 0.19) (Fig. S5A and Table S2). For the regional association analysis with the lung disease phenotype of forced expiratory volume in 1 s (FEV_1_) (*n *= 1,600) (31), no SNP reached regional significance, and there was no evidence of association at rs10875080 or rs11582736 (Fig. S5B and Table S2). Thus, our results confirm the translational relevance of murine genetic mapping for human respiratory infection by P. aeruginosa.

10.1128/mBio.00097-20.5 FIG S4 Distribution of protein-coding genes located in the murine quantitative trait locus and the homologous conserved human genomic region. Shown are protein-coding genes of the quantitative trait locus present in the syntenic genomic block between mouse (A) and human (B) genomes. Download FIG S4, TIF file, 0.5 MB. Copyright © 2020 Loré et al. 2020 Loré et al. This content is distributed under the terms of the Creative Commons Attribution 4.0 International license.

10.1128/mBio.00097-20.6 FIG S5 Genetic association analysis at a human QTL. (A) Association analysis between the square-root-transformed age at chronic P. aeruginosa infection and SNPs located at the human QTL in 256 Canadian CF patients of European descent (chromosome 1 Mb 97.0 to 105.0 [17,270 SNPs were tested]; human genome version 19). (B) Analysis of the association of forced expiratory volume in 1 s (FEV_1_)-based lung function phenotypes and SNPs located at the human QTL in 1,600 Canadian CF patients of European descent (Chr 1 Mb 97.0 to 105.0 [16,999 SNPs were tested]; human genome version 19). Download FIG S5, TIF file, 2.6 MB. Copyright © 2020 Loré et al. 2020 Loré et al. This content is distributed under the terms of the Creative Commons Attribution 4.0 International license.

10.1128/mBio.00097-20.9 TABLE S2 Characteristics of patients included in this study and genetic analyses. Download Table S2, DOCX file, 0.03 MB. Copyright © 2020 Loré et al. 2020 Loré et al. This content is distributed under the terms of the Creative Commons Attribution 4.0 International license.

## DISCUSSION

Investigation of host genetic factors that modify susceptibility to infection is important for our understanding of disease processes (32, 33). We have demonstrated in CC lines distinct disease phenotypes, ranging from complete resistance to severe pneumonia, confirming the influence of the host genetic background on the response to P. aeruginosa infection. On the basis of these results, we identified a QTL on murine Chr 3 that modified the severity of respiratory infections. Prioritization of candidate modifier genes within the QTL led us to select the *S1pr1* gene for functional validation of its relevance for respiratory infection. This gene encodes a G-protein-coupled receptor with a fundamental physiological role in inflammation, mediating immune cell trafficking. S1P1 is expressed in a variety of cell types, including endothelial and epithelial cells, T cells, natural killer T cells, innate lymphoid cells, and dendritic cells (26, 34, 35). In humans, 15 nonsynonymous *S1PR1* SNPs have been identified by the U.S. National Heart, Lung, and Blood Institute Exome Sequencing Project (36). Functional variants have been associated with asthma susceptibility, constituting a risk factor for disease severity (37), which supports the idea that genetic variants of *S1PR1* may influence physiological and pathological events in the lung compartment. Our work has not identified genetic variants of *S1PR1* associated with the severity of P. aeruginosa respiratory infection in the Canadian CF Gene Modifier cohort; however, we cannot exclude the presence of nonsynonymous *S1PR1* SNPs in different cohorts of patients, including different respiratory diseases (e.g., COPD). It is worth noting that, at a functional level, our results suggest that modulation of S1P1 modifies the outcome of infectious processes, at least in our *in vivo* model of respiratory infection. For the functional-validation approach by pharmacological targeting, C57BL/6NCrl mice were chosen based on their intermediate susceptibility to infection with the P. aeruginosa AA2 strain (∼25 to 40% survival rate) (17, 32), giving us the opportunity to evaluate beneficial or detrimental effects of treatment. Using the Ex26 compound and the FTY720 antagonist, we demonstrated that S1P1 modulation is relevant for the outcome of P. aeruginosa infection. Although it is possible that FTY720 might also act on other S1P receptors, such as S1P3, S1P4, and S1P5, the Ex26 compound is a selective S1P1 receptor antagonist, confirming the relevance of this receptor in the outcome of P. aeruginosa infection. Notably, treatment with either Ex26 or FTY720 results in alteration of trafficking and egress of lymphocytes (27, 38), and lymphocyte egress is mediated by S1P1 activity (39). In addition, in another study, sphingosine was involved in pulmonary infection in mice with burn injuries (40), suggesting that S1P1 may also have a role during P. aeruginosa infection processes, as indicated by our results.

Our QTL mapping results with CC mice enabled us to focus attention on a small syntenic human genetic locus, thereby gaining statistical power since only a small genomic region needed to be tested for association. This approach yielded genetic modifiers potentially relevant to human lung disease, even using relatively small sample sizes and/or limited phenotyping. Assuming that P. aeruginosa respiratory infections are associated with a wide range of pathological and clinical phenotypes, owing to different etiologies and conditions (e.g., CF and COPD), the availability of phenotyped and genotyped cohorts of patients is limited. The cohort of patients with CF that we investigated has been previously genotyped and characterized in a candidate gene study for the development of P. aeruginosa infection (30). At the syntenic human locus on chromosome 1, intronic genetic variation in *DPYD* was associated with age at the first P. aeruginosa infection. It is worth noting that *DPYD* also showed the highest genome-wide significance in the murine QTL peak. *DPYD* encodes dihydropyrimidine dehydrogenase, which catalyzes the rate-limiting step in the catabolic pathway of uracil and thymidine. *DPYD* deficiency has wide-ranging implications, and genetic variation in *DPYD* is associated with the risk of severe toxic reactions to fluoropyrimidines, which are used to treat cancer (41). Studies of genotype-phenotype correlation related to *DPYD* are limited, but results have demonstrated that *DPYD* is required for the epithelial-to-mesenchymal transition (42), suggesting involvement in lung disease via pulmonary fibrosis. The location of the associated SNPs in a noncoding region of *DPYD* suggests that they may affect the gene regulation of either DPYD or possibly other *cis* or *trans* genes. The involvement of DPYD in metabolic pathways suggests the possibility of a role in immunological mechanisms. Functional studies will be required to assess the role of *DPYD* in host defense against infection and to determine the effects of the SNPs on the regulation of gene expression and on DPYD activity. Our data suggest that genetic variation in *DPYD* is relevant to P. aeruginosa infection as a potential human biomarker, although the effects of the identified variants on *DPYD* or on other genes in the identified human locus require further investigation.

The translational nature of our experimental approach provides a proof of concept that could be exploited for other human pathologies mediated by bacterial infection, using natural variation in mouse models to prioritize human candidate genes. Our results have demonstrated that *in vivo* models of infections integrated with parallel human studies are useful for making new discoveries in complex human disease.

## MATERIALS AND METHODS

### Ethics statement.

Animal studies were approved by the Institutional Animal Care and Use Committee of Tel Aviv University (TAU) and the San Raffaele Scientific Institute (Milan, Italy). Human studies for mapping and isolation of genes influencing the severity of disease in CF were approved by the SickKids Research Ethics Board, Toronto, Ontario, Canada. The P. aeruginosa clinical isolate AA2, derived from a patient with CF attending the Medizinische Hochschule in Hannover, Germany, was previously described (16, 17, 43, 44). Research on the bacterial isolates from the individual with CF was approved by the responsible physician at the CF center at Hannover Medical School, Germany, and the patient gave informed consent before sample collection. Approval for the storage of biological materials was obtained by the Hannover Medical School, Germany.

### Collaborative Cross and inbred murine lines.

A total of 221 CC mice (100 female and 121 male) from 39 lines (average of 5 mice per CC line), 7 to 14 weeks old, were used. Full details of the development of the CC colony have been previously described (9, 45) (https://csbio.unc.edu/CCstatus/index.py). Initial genotyping was conducted on CC mice at generations of between 15 and 20 (>80% homozygosity), and the mice that were used in the study were at least 6 to 8 generations later, which corresponds to 96 to 99.9% genetic homozygosity.

A/J mice, purchased from Jackson Laboratory USA, were used as internal controls for each batch of experiments with CC mice. C57BL/6NCrl male mice, purchased from Charles River (Lecco, Italy), 8 to 9 weeks old, were used for treatment with the Ex26 and FTY720 molecules.

### Mouse model of acute P. aeruginosa infection.

Mice were infected by intratracheal (i.t.) injection of P. aeruginosa AA2 as previously described (16, 17, 43, 46). Prior to animal experiments, the P. aeruginosa AA2 clinical strain was grown for 3 h in Trypticase soy broth (TSB) (BD [Becton and Dickinson]) at 37°C to reach the exponential phase (16, 17, 43, 46). The bacteria were pelleted by centrifugation (2,700 × *g* for 15 min at 4°C), resuspended in sterile phosphate-buffered saline (PBS), and diluted to give the required dose in 60 μl. Mice were anesthetized by an intraperitoneal (i.p.) injection of a solution of Avertin (2,2,2-tribromethanol) (97%) in 0.9% NaCl administered at a volume of 0.015 ml/g of body weight and then infected i.t. with a dose of 1 × 10^6^ or 5 × 10^6^ CFU of P. aeruginosa, as described previously (16, 17, 43). Survival times and body weights of mice were recorded daily over 1 week. In addition, mice were monitored for coat quality, posture, attitude, ambulation, and hydration status. Mice that lost >25% of their body weight and had evidence of severe clinical disease were sacrificed before the termination of the experiments, with an overdose of carbon dioxide. Whole blood was collected and analyzed immediately. Hematocrit was carried out with an automated ProCyte Dx hematology analyzer (Idexx BioResearch, USA).

### Estimation of heritability.

Broad-sense heritability, including epistatic but not dominance effects for survival time, was calculated across the CC lines under P. aeruginosa infection, as previously described (16).

### QTL data analysis.

Data analysis was performed with R statistical software (R Development Core Team, 2009), including the R package HAPPY.HBREM (9). Survival data for the CC lines were converted into binary alive/dead phenotypes, one for each day of the trial. These binary phenotypes were analyzed for the presence of QTLs by a two-stage process. First, a logistic regression model was used to fit covariates (gender, batch, and initial body weight) with the R function glm(); next, the residuals from the model were used as the response variable for QTL mapping by linear regression, with the Bayesian random-effects model HBREM, to estimate individual haplotype effects. One covariate (initial body weight) significantly affected survival, while gender and batch effects did not. The mean values of residuals across replicates within each CC line were used in the QTL analysis. The presence of a QTL was tested by analysis of variance (ANOVA) by comparing the fit of the genetic model with the null hypothesis. QTL mapping with CC strains consists of eight-way haplotype linear regression with an additive model as described previously (9). Genome-wide significance was estimated by permutation (1,000), where the CC line labels were permuted between the phenotypes (9, 15, 18). QTL effect sizes were estimated as the proportion of the log likelihood explained by the locus effects at the QTL. Genome-wide significance (at 50%, 10%, and 5% genome-wide significance levels) was estimated by permuting the CC strains (1,000 tests). Additional details are provided in Text S1 in the supplemental material.

10.1128/mBio.00097-20.1 TEXT S1 Supplemental methods. Download Text S1, DOCX file, 0.04 MB. Copyright © 2020 Loré et al. 2020 Loré et al. This content is distributed under the terms of the Creative Commons Attribution 4.0 International license.

### Testing sequence variation segregating between the CC founders.

Merge analysis (9) was utilized to test which variants under a QTL peak were compatible with the pattern of action at the QTL. Sequence variants were identified from the Sanger Institute Mouse Genomes Project database (47).

### Mouse treatment with Ex26 and FTY720.

The S1P1 receptor antagonist compound Ex26 (catalogue number 5833; Tocris Bioscience) and the S1P analogue FTY720 (catalogue number SML0700; Sigma-Aldrich, Milan, Italy) were prepared in 3% dimethyl sulfoxide (DMSO) (Sigma-Aldrich, Milan, Italy). First, C57BL/6NCrl mice were treated with Ex26 (3 mg/kg of body weight) and FTY720 (3 mg/kg), administered i.p., to test the toxicity of the molecules. Control mice were injected with 3% DMSO in sterile water. Next, C57BL/6NCrl mice were infected with P. aeruginosa by i.t. administration and treated with Ex26 (3 mg/kg), FTY720 (3 mg/kg), or the vehicle (3% DMSO) in a volume of 100 μl by i.p. administration. Additional control mice were challenged with PBS by i.t. administration and treated with Ex26 or FTY720 as described above. In all mice, Ex26, FTY720, or 3% DMSO treatment started 2 h before P. aeruginosa infection or PBS challenge, followed by treatment twice a day for 7 days. Survival was monitored twice a day.

### Databases and bioinformatics tool for gene prioritization in the QTL.

SNPs were identified from the Sanger Institute Mouse Genomes Project database (47). Protein expression in the respiratory system was identified from the Human Protein Atlas Database (24). Prioritization of candidate modifier genes was performed with the Beegle online tool (25) using a list of training genes obtained with the query “lung infection” (Genes_List_Query_Lung Infection.csv). Synteny Portal was used as a tool to perform comparative genomics studies and syntenic analysis (http://bioinfo.konkuk.ac.kr/synteny_portal/).

### Human subjects, phenotypes, and genotypes.

The primary phenotype analyzed for association in the Canadian CF Gene Modifier cohort was the age at the first infection with P. aeruginosa (*n* = 712). Follow-up analysis evaluated the association evidence at the locus with the related phenotypes of lung disease severity (*n* = 1,600), which is calculated as a function of FEV_1_ (31), and age at chronic P. aeruginosa infection (*n* = 256). Chronic P. aeruginosa infection was defined as three consecutive encounters of positive P. aeruginosa infections at any point in the 3-year window in a patient’s clinical record. Not all patients in the Canadian Cystic Fibrosis Gene Modifier Study (GMS) have complete data on all three phenotypes analyzed in this paper, which explains the different sample sizes in each analysis. The 1,600 samples analyzed for association with lung disease are samples from the Canadian Cystic Fibrosis GMS that were included in a previous CF lung GWAS study (48). The 712 subjects analyzed for age at the first infection are individuals from the same GMS study who had P. aeruginosa infection status measured, with only 570 individuals overlapping the 1,600 individuals in the study by Corvol et al. (48). The 256 individuals analyzed for age at chronic P. aeruginosa infection is a subset of the 712 individuals who had the appropriate longitudinal data to assess whether there was chronic P. aeruginosa infection. Patient characteristics are reported in Table S2A in the supplemental material.

### Estimation of human population structures by principal-component analysis.

The KING package was used to obtain principal components to serve as covariates to adjust for population structure, accounting for related individuals. Input variables for the analysis were the genotypes of SNPs with a high minor allele frequency and low linkage disequilibrium. The Tracy-Widom statistic, computed by EIGENSOFT (49), was used to evaluate the significance of each principal component (Table S2B). For each individual in the CF cohort analyzed, the values for the first three principal components were plotted, along with those for HapMap3 samples. Where individuals from the CF population overlapped HapMap3 groups other than European and Mexican groups, they were excluded from the association analysis (Fig. S6).

10.1128/mBio.00097-20.7 FIG S6 Estimation of human population structures by principal-component analysis. The principal-component analysis excluded patients from the Canadian CF cohort who do not cluster with European populations. Six patients who were identified as Indians were removed from the study (black dots). Admixed individuals were included in the analysis, and their admixture was adjusted for by using the significant principal components. Download FIG S6, TIF file, 0.2 MB. Copyright © 2020 Loré et al. 2020 Loré et al. This content is distributed under the terms of the Creative Commons Attribution 4.0 International license.

### Association testing at the human locus.

Association analysis was conducted between SNPs at the Chr 1 region spanning Mb 97.0 to 105.0 (hg19) and infection phenotypes (age at first P. aeruginosa infection, age at chronic P. aeruginosa infection, and lung function severity [31]). SNPs with a minor allele frequency of >5% were included and were coded additively in the model. The age at the first P. aeruginosa infection and the age at chronic P. aeruginosa infection were square-root transformed. All association analyses used generalized estimating equations with an identity link implemented in the R statistical package, accounting for sibling correlations with the exchangeable covariance structure. Sex and significant principal components were included in the model as covariates for each SNP. To account for multiple-hypothesis testing, the *P* value threshold for significance was calculated with the GEC package (50), based on the effective number of independent SNPs analyzed at the locus (Table S2).

### Data availability.

All the data used for the QTL analysis are available in Data Set S1 in the supplemental material. The data and software used are available at http://mtweb.cs.ucl.ac.uk/mus/www/preCC.
