# Supplementary material for: Collaborative Cross Mice Yield Genetic Modifiers for Pseudomonas aeruginosa Infection in Human Lung Disease
Source: mBio. 2020 Mar 3;11(2):e00097-20. doi: 10.1128/mBio.00097-20 (PMC7064750; doi:10.1128/mBio.00097-20)
Supplement: TABLE S1 [file mBio.00097-20-st001.docx]

**Table S1. Prioritization of candidate genes for susceptibility to *P. aeruginosa* respiratory infection.**

Prioritization of protein-coding genes identified in the mouse Chr 3 QTL that is associated with survival of *P. aeruginosa* infection. Genes were assigned points according to genome-wide significance of their location within the QTL and expression of proteins in lung pneumocytes and bronchial cells (according to information in the Human Protein Atlas Database Version 18). Genes were ranked according to the total number of points assigned to each.

| **Gene name** | **Gene localization** | **E**  **<0.05** | **E**  **<0.1** | **E**  **<0.5** | **Expression** | | | **Score** |
| --- | --- | --- | --- | --- | --- | --- | --- | --- |
|  |  |  |  |  | **Pneumocytes** | **Bronchus** | |  |
| *Dpyd* | chr3(+):118265047–119135836 | 1 | 1 | 1 | 1 | | 1 | 5 |
| *Ptbp2* | chr3(-):119421660–119486306 | 1 | 1 | 1 | 1 | | 1 | 5 |
| *Snx7* | chr3(-):117484415–117571854 |  | 1 | 1 | 1 | | 1 | 4 |
| *S1pr1* | chr3(-):115413351–115417973 |  |  | 1 | 1 | | 1 | 3 |
| *Col11a1* | chr3(+):113733458–113923636 |  |  | 1 | 1 | | 1 | 3 |
| *Dph5* | chr3(+):115591083–115637279 |  |  | 1 | 1 | | 1 | 3 |
| *Vcam1* | chr3(-):115812938–115832606 |  |  | 1 | 1 | | 1 | 3 |
| *Cdc14a* | chr3(-):115975471–116126950 |  |  | 1 | 1 | | 1 | 3 |
| *Dbt* | chr3(+):116215988–116252894 |  |  | 1 | 1 | | 1 | 3 |
| *Lrrc39* | chr3(+):116265891–116286052 |  |  | 1 | 1 | | 1 | 3 |
| *Agl* | chr3(-):116442917–116511084 |  |  | 1 | 1 | | 1 | 3 |
| *Frrs1* | chr3(+):116581145–116606668 |  |  | 1 | 1 | | 1 | 3 |
| *Palmd* | chr3(-):116621174–116671905 |  |  | 1 | 1 | | 1 | 3 |
| *Gpr88* | chr3(-):115952572–115956402 |  |  | 1 | 1 | | 1 | 3 |
| *Slc30a7* | chr3(-):115641891–115710324 |  |  | 1 | 1 | | 1 | 3 |
| *Extl2* | chr3(+):115710380–115731934 |  |  | 1 | 1 | |  | 2 |
| *Rtcd1* | chr3(-):116191881–116211126 |  |  | 1 |  | | 1 | 2 |
| *Trmt13* | chr3(-):116284011–116317505 |  |  | 1 | 1 | |  | 2 |
| *Slc35a3* | chr3(-):116372387–116415749 |  |  | 1 |  | | 1 | 2 |
| *4833424O15Rik* | chr3(+):117278145–117392425 |  | 1 | 1 |  | |  | 2 |
| *Rnpc3* | chr3(-):113307985–113333067 |  |  | 1 |  | |  | 1 |
| *Sass6* | chr3(+):116297926–116333902 |  |  | 1 |  | |  | 1 |
| *Hiat1* | chr3(-):116334082–116384178 |  |  | 1 |  | |  | 1 |
| *Plppr4* | chr3(-):117022063–117063794 |  |  | 1 |  | |  | 1 |
| *Amy1* | chr3(-):113258871–113280748 |  |  | 1 |  | |  | 1 |
| *Olfm3* | chr3(+):114606996–114828640 |  |  | 1 |  | |  | 1 |
| *Amy2a2* | chr3(-):113149973–113235208 |  |  | 1 |  | |  | 1 |
| *Amy2a1* | chr3(-):113232321–113235346 |  |  | 1 |  | |  | 1 |
| *Amy2a5* | chr3(-):113052094–113061755 |  |  | 1 |  | |  | 1 |
| *Amy2a4* | chr3(-):113084761–113094367 |  |  | 1 |  | |  | 1 |
| *Amy2a3* | chr3(-):113117373–113159438 |  |  | 1 |  | |  | 1 |
