# Supplementary material for: Collaborative Cross Mice Yield Genetic Modifiers for Pseudomonas aeruginosa Infection in Human Lung Disease
Source: mBio. 2020 Mar 3;11(2):e00097-20. doi: 10.1128/mBio.00097-20 (PMC7064750; doi:10.1128/mBio.00097-20)
Supplement: TABLE S2 [file mBio.00097-20-st002.docx]

**Table S2. Characteristics of patients included in this study and genetic analysis.**

**A) Characteristics of patients included in this study.**

In this study we used the already genotyped Canadian CF Gene Modifier cohort with clinical microbiological data for *P. aeruginosa* infection previously collected ([4](#_ENREF_4)).

| Sample size | 712 |
| --- | --- |
| Number of independent families | 669 |
| Number of females | 357 (49.7%) |
| Age at first *P. aeruginosa* infection (years) | Min: 0.1 |
|  | 25%: 2.1 |
|  | Median: 5.5 |
|  | 75%: 9.1 |
|  | Max: 20.5 |
|  | Mean: 6.0 |

**B)Assessment of disease-modifier genes in a cohort of CF patients.**

**Tracy–Widom test for significance (*P* <0.05) of principal components.**

| No. | Eigenvalue^$^ | Difference^%^ | Twstat^#^ | *P* *value^&^* |
| --- | --- | --- | --- | --- |
| **1** | **112.69** | **NA** | **8.285** | **1.03E−08** |
| **2** | **108.79** | **−3.9** | **10.888** | **3.07E−12** |
| **3** | **102.65** | **−6.14** | **10.111** | **3.89E−11** |
| 4 | 97.3 | −5.35 | −0.261 | 0.220315 |
| 5 | 96.7 | −0.6 | −1.479 | 0.567153 |

^$^ The first five eigenvalues of principle component analysis. ^%^ Difference between the last two eigenvalues. ^#^ Test statics for Tracy-Widom (TW) test. *^&^* P-value of Tracy-Widom test for significance of principle components

**C) Association test for confounders in a model for the age of first *P. aeruginosa* infection**.

|  | Coefficient | StdErr^%^ | Wald Chi-sqr^#^ | *P value* |
| --- | --- | --- | --- | --- |
| SEX | −0.0296 | 0.0700 | 0.1780 | 0.6728 |
| PC1^$^ | −0.3683 | 1.0753 | 0.1170 | 0.7319 |
| PC2^$^ | −2.7720 | 0.9673 | 8.2130 | 0.0042 |
| PC3^$^ | 0.1609 | 0.8313 | 0.0370 | 0.8465 |

^$^ Significant principle components (PC) (*P*<0.05 in TW test) with the largest (second and third largest) eigenvalue. ^%^ Standard error for estimated coefficient. ^#^ Wald Chi-square statistics.

**D) Significance criteria for regional association analysis based on the effective number of independent tests in the region (GEC calculator (**[**5**](#_ENREF_5)**))**

| Tests | Observed_  number | Effective_  number | Suggestive_*P*_value | Significant_*P*_value | Highly_significant_*P*_value |
| --- | --- | --- | --- | --- | --- |
| Age at first *P. aeruginosa* infection | 17139 | 3150.6 | 3.17E−04 | **1.59E−05** | 3.17E−07 |
| Age at chronic *P. aeruginosa* infection | 17270 | 3024.89 | 3.31E−04 | **1.65E−05** | 3.31E−07 |
| Forced expiratory volume in 1 s | 16999 | 3234.57 | 3.09E−04 | **1.55E−05** | 3.09E−07 |

**E) Test statistics for the two DPYD markers associated with age at first *P. aeruginosa* infection.**

|  | **Coeffecient** | **Std.Err.** | **p-value** |
| --- | --- | --- | --- |
| *Age at the first P. aeruginosa infection (n=712)* | | | |
| rs10875080 | 0.255 | 0.057 | 6.73E-06 |
| rs11582736 | 0.254 | 0.059 | 1.56E-05 |
| *Age at chronic P. aeruginosa infection (n=256)* | | | |
| rs10875080 | 0.19 | 0.08 | 0.02 |
| rs11582736 | 0.16 | 0.08 | 0.06 |
| *Lung-disease phenotype (n=1600)* | | | |
| rs10875080 | -0.00090 | 0.03 | 0.98 |
| rs11582736 | 0.00384 | 0.04 | 0.92 |
