# Supplementary material for: Collaborative Cross Mice Yield Genetic Modifiers for Pseudomonas aeruginosa Infection in Human Lung Disease
Source: mBio. 2020 Mar 3;11(2):e00097-20. doi: 10.1128/mBio.00097-20 (PMC7064750; doi:10.1128/mBio.00097-20)
Supplement: TEXT S1 [file mBio.00097-20-s0001.docx]

**Collaborative Cross mice yield genetic modifiers for *Pseudomonas aeruginosa* infection in human lung disease**

Nicola Ivan Loré, Barbara Sipione, Gengming He, Lisa J Strug, Hanifa J Atamni, Alexandra Dorman, Richard Mott, Fuad A. Iraqi, Alessandra Bragonzi

Supplementary Methods

**Supplementary Methods**

**CC lines**

Mice of the CC lines were provided by the Small Animal Facility at Sackler Faculty of Medicine, TAU where 3-5 mice per cage were housed. Mice were housed on hardwood chip bedding in ventilated cages, maintained at a 12:12‐h light:dark cycle at a temperature of 21‐23°C. The mice were given tap water and standard rodent chow diet ad libitum, which consists of %Kcal from 18% fat, 24% protein and 58% carbohydrates (TD.2018SC; Teklad Global, Harlan, Madison, WI, USA), since weaning at the age of 3 weeks until the age of 10 weeks. Mice were monitored twice a day for coat quality, posture, attitude, ambulation and hydration status. Body weight was recorded daily in the late morning. Mice were tested for pathogens following FELASA recommendation ([www.felasa.eu](http://www.felasa.eu)).

**QTL data analysis.**

Data analysis was performed with the statistical software R (R Development Core Team 2009), including the R package HAPPY.HBREM ([1](#_ENREF_1)). Survival data for the CC lines were converted into binary alive/dead phenotypes, one for each day of the trial. These binary phenotypes were analysed for the presence of QTLs by a two-stage process. First, a logistic regression model was used to fit covariate (initial body weights) with the R function glm(), then the residuals from the model were used as the response variable for QTL mapping by linear regression, with the Bayesian random effects model HBREM, to estimate individual haplotype effects. Covariates (gender and age) did not significantly affect survival (data not shown). The final logistic regression model with alive/dead status *y* was:

$$ln\frac{\pi(y)}{1-\pi(y)}= \mu$$

where *π*(*y*) is the probability of being dead, and *μ* is the intercept.

The genome of each CC line is a mosaic derived from the inbred founders, which we reconstructed using a hidden Markov Model (HAPPY) across the genotypes, to compute probabilities of descent from the founders. In CC line *k* at SNP interval (locus) *L*, the probability of descent from founder strain *s* was denoted by *P_LK_*(*s*). The presence of a QTL at *L* was tested with a linear regression framework, in which the residual deviance from the mean probability of death *y_k_* for an individual from line *k* was represented in the following formula:

$$ln\frac{\pi(y_{k})}{1-\pi(y_{k})}\mu+\sum_{s} P_{LK} \left( s \right)\beta_{s}$$

where *μ* is the overall mean and *βs* is the effect of founder haplotype *s* at *L*. The presence of a QTL was tested by comparing the fit of the model with that of a simpler submodel in which *βs* = 0 (the null hypothesis). Significance was reported as log*P*, the negative log_10_ of the *P* value of the test of the null hypothesis, as computed by the R anova() function. Genome-wide significance was estimated by permutation (1000), where the CC line labels were permuted between the phenotypes ([1-3](#_ENREF_1)). The mean probability of death across replicates within each CC line was used in the QTL analysis. QTL effect sizes were estimated as the proportion of the log likelihood explained by the locus effects at the QTL ([1-3](#_ENREF_1)). The survival analysis plot was produced by first selecting the chr 3 QTL peak at 7 Days (near SNP JAX00112378, or rs30212372), and assigning to each CC line its most probable founder strain based on the HAPPY probabilities of descent from each of the eight founders. The survival plot was then generated using the R command “> ggsurvplot( s ~ founder )” where s is the Surv() object for the data, and founder is a factor indicating which founder strain was present.

**References**

1. Durrant C, Tayem H, Yalcin B, Cleak J, Goodstadt L, de Villena FP, Mott R, Iraqi FA. 2011. Collaborative Cross mice and their power to map host susceptibility to Aspergillus fumigatus infection. Genome Res 21:1239-48.

2. Abu-Toamih Atamni HJ, Ziner Y, Mott R, Wolf L, Iraqi FA. 2017. Glucose tolerance female-specific QTL mapped in collaborative cross mice. Mamm Genome 28:20-30.

3. Vered K, Durrant C, Mott R, Iraqi FA. 2014. Susceptibility to klebsiella pneumonaie infection in collaborative cross mice is a complex trait controlled by at least three loci acting at different time points. BMC genomics 15:865.

4. Li W, Soave D, Miller MR, Keenan K, Lin F, Gong J, Chiang T, Stephenson AL, Durie P, Rommens J, Sun L, Strug LJ. 2014. Unraveling the complex genetic model for cystic fibrosis: pleiotropic effects of modifier genes on early cystic fibrosis-related morbidities. Hum Genet 133:151-61.

5. Price AL, Patterson NJ, Plenge RM, Weinblatt ME, Shadick NA, Reich D. 2006. Principal components analysis corrects for stratification in genome-wide association studies. Nat Genet 38:904-9.
